# Neural Processes Underlying the“Same”-“Different” Judgment of Two Simultaneously Presented Objects- An EEG Study

**DOI:** 10.1371/journal.pone.0081737

**Published:** 2013-12-12

**Authors:** Ruiling Zhang, Zhonghua Hu, Roberson Debi, Lingcong Zhang, Hong Li, Qiang Liu

**Affiliations:** 1 School of Psychology, Liaoning Normal University, Dalian, China; 2 Department of Psychology, University of Essex, Wivenhoe Park, Colchester, Essex, United Kingdom; 3 Department of Educational Science and Technology, Minnan Normal University Zhangzhou, China; Cuban Neuroscience Center, Cuba

## Abstract

The present study investigated the neural processes underlying “same” and -“different” judgments for two simultaneously presented objects, that varied on one or both, of two dimensions: color and shape. Participants judged whether or not the two objects were “same” or “different” on either the color dimension (color task) or the shape dimension (shape task). The unattended irrelevant dimension of the objects was either congruent (same-same; different-different) or incongruent (same-different). ERP data showed a main effect of color congruency in the time window 190–260 ms post-stimulus presentation and a main effect of shape congruency in the time window 220–280 ms post-stimulus presentation in both color and shape tasks. The interaction between color and shape congruency in the ERP data occurred in a later time window than the two main effects, indicating that mismatches in task-relevant and task-irrelevant dimensions were processed automatically and independently before a response was selected. The fact that the interference of the task-irrelevant dimension occurred after mismatch detection, supports a confluence model of processing.

## Introduction

“Sameness and difference are fundamental cognitive relations that enter, at least implicitly, into most forms of adaptive perceptual behavior.” [1].

Requiring subjects to judge two stimuli as “same” or “different” with respect to their similarities and differences on some criterion (SDJ) is one of the most familiar experimental paradigms used to investigate human information processing [1], [2]. The SDJ task has many different permutations [1], but the following study focuses on one particular version of this task. Specifically, we focus on SDJ judgments for two simultaneously presented objects that differ on various dimensions such as shape or color. Although the objects could differ on task-relevant dimension as well as task-irrelevant dimensions, participants were required to make a “same”- “different” judgment only on the task-relevant dimension and were not explicitly told about the task-irrelevant (unattended) dimension.

At a behavioral level, this SDJ paradigm has consistently shown that changes on the irrelevant dimension affect performance on the relevant dimension [3]–[8]. Yet it remains unclear how the pattern of behavioral results maps on to specific mechanisms and processes at the neural level. Two models (a confluence model and a response competition model) have been proposed to explain the behavioral findings at the level of neural processes, but the evidence from empirical studies for either of these models remains inconclusive. In particular, there is a lack of electrophysiological (EEG) evidence in the specific case of making “same”-“different” judgments for two simultaneously presented objects. The present EEG study aimed to empirically test the neural processes underlying “same”-“different” judgments about two simultaneously presented objects when both task-relevant and task-irrelevant dimensions were manipulated.

Early behavioral studies of this SDJ paradigm found that reaction times (RTs) on the task-relevant dimension were modulated by whether or not the objects were same or different in the task-irrelevant dimension [4], [5], [7]–[9]. Participants took longer to make “same” judgments on the shape dimension when the objects differed on the task-irrelevant dimension - color, compared to when they were of the same color. Similarly, “different” judgments on shapes of two objects were faster when the objects also differed in color compared to when they were the same color.

Two models have been proposed to explain these behavioral results at a neural level. Eviatar et al. suggested a confluence model [12], based on the discrete-stage theory [10], [11]. The confluence model posits that objects are compared automatically with respect to the task-relevant and task-irrelevant dimensions via separate neural systems before the outputs converge to a point of confluence where they affect the final judgment. When outputs of the processing of task-relevant and task-irrelevant dimensions are in accordance, i.e. congruent, the response to that trial would be facilitated. However, if the two independently computed outputs are incongruent, the response would be inhibited and slower. According to this model, the comparison of objects on the task-relevant dimension is completed before selective response activation commences, with no temporal overlap between the two stages.

Alternatively, Eriksen et al. suggested a response competition model [3], based on the concept of continuous flow [13]. This model posits that visual perception occurs gradually. As a percept develops, the similarities and differences between the stimuli on each dimension prime the relevant response in parallel, as soon as they are detected and regardless of whether or not they are task-relevant. The response would be produced as soon as the relevant response reaches threshold criterion. If the task-irrelevant dimension is incongruent with the task-relevant dimension, information from the task-irrelevant dimension would simultaneously prime a competing response. Partial activation of the competing response option would delay the execution of the correct response. According to this model, response activation starts as soon as information associated to the responses has become available and before comparison of the objects is complete.

Although these two models differ in regard to the processing stage at which the response begins to be activated, both models suggest that the task-relevant and task-irrelevant dimensions are computed automatically and independently at a neural level.

To date, several EEG studies have attempted to pin down the extent to which these processes are automatic and independent by analyzing high temporal resolution event-related potentials (ERPs) to reveal underlying neural mechanisms. Those studies have examined the processes by presenting the pairs of stimuli in a sequential order, rather than simultaneously, and consistently observed a late negative ERP component N270 when two different stimuli were shown sequentially [14]–[21]. The N270 component has been found for mismatches between S2 and S1 on color [14], [18], [22], shape [14], [22]–[24], orientation [16], position [21], [25], or digit value [15], and was not influenced by stimulus probability [14]. It was therefore suggested that the N270 represented neural activity relating to detection of mismatch in the information presented [14], [15], [17], [26].

However, these studies cannot distinguish whether or not detection of a mismatch between objects on the task-relevant and task-irrelevant dimensions occurs via separate processes in an automatic and independent manner. On the one hand, the N270 also emerged when the stimuli differed only on the task-irrelevant dimension, albeit with a smaller amplitude than if the objects differed on the task-relevant dimension [21], [22], indicating that mismatched information on the task-irrelevant dimension was also automatically processed. This would be compatible with detection of a mismatch on both task-relevant and task-irrelevant dimensions occurring automatically and independently. On the other hand, Wang et al. (2004) reported that the amplitude of the N270 elicited by pairs of sequentially presented objects differing on both task-relevant and task-irrelevant dimensions was no larger than that elicited by a mismatch on the task-relevant dimension alone [22]. Such a finding challenges the assumption that mismatched information from task-relevant and task-irrelevant dimensions is processed independently, since automatic independent processing should result in both dimensions eliciting independent overlapping N270 components, cumulatively larger than that elicited by a mismatch on either single dimension.

These contradictory ERP findings could be due to sequential stimulus presentation. One problem for this paradigm is that encoding of the second stimulus necessarily begins later than encoding of the first, and would thus be compared to already encoded information about the first stimulus (which would need to be retrieved from memory). While processing the second stimulus, in order to minimize cognitive load, participants may only store information about the task-relevant dimension of the first stimulus, leading to less task-irrelevant information being available. This would lead to the lack of a significant interaction between the task-relevant and the task-irrelevant dimension in a sequential SDJ task. Another problem for this paradigm is that sequential presentation necessarily involves memory and has been shown to rely heavily on verbal labels rather than visual comparisons [27], [28], with the consequence that easy-to-name dimensions (such as basic color categories) are recalled better than hard-to-name dimensions (such as orientation) regardless of the physical differences between stimuli on each dimension.

In contrast, in a simultaneous SDJ task, information from both stimuli should be processed and compared at the same time, so there should be no selective loss of information and the congruency of the task-irrelevant dimension should interfere with judgments about the task-relevant dimension.

**Figure 1:**
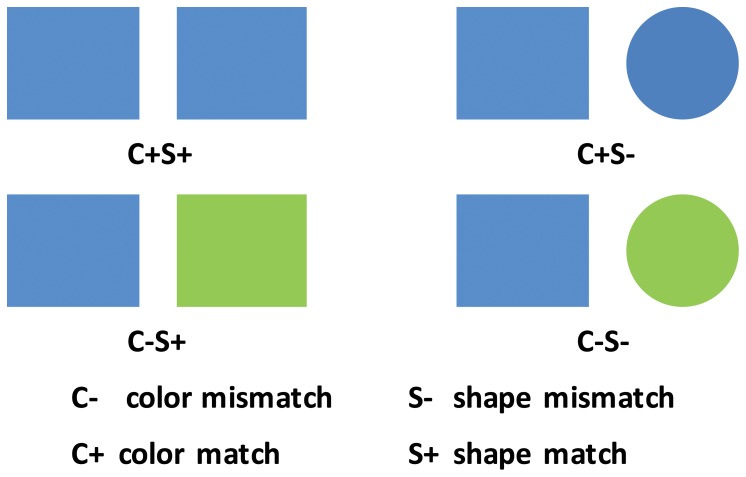
Examples of stimulus pairs used in the experiment. C: color, S: shape,+indicates a match, - indicates a mismatch.

The purpose of the present study was therefore to build on previous ERP studies and investigate the neural mechanisms involved in processing the classical simultaneous SDJ behavioral task. Specifically, we aimed to replicate previous SDJ behavioral findings at the same time as gathering data at the neural level, in order to establish whether the “same”-“different” judgment on the task-relevant dimension for two simultaneously presented objects is affected by the presence of additional information about these two objects on a task-irrelevant dimension (Figure 1).

From previous behavioral studies, we expected that RTs would be shorter when the objects’ relationship on the task-relevant dimension (“same” or “different”) was congruent with their relationship on the task-irrelevant dimension compared to when the task-relevant and task-irrelevant dimension were incongruent. At the ERP level two objects that matched on only one dimension (either color or shape) but not the other, should elicit a significantly more negative potential 200–300 ms post-stimulus presentation than two objects that matched on both dimensions, irrespective of whether or not the mismatch occurred in the task-relevant dimension.

Furthermore, early emergence of congruency effects for both attended and unattended dimensions (200–300 ms post stimulus) would indicate that mismatch detection occurs automatically and in parallel regardless of task set. We also hypothesized that the response judgment stage would be the point at which reactions to attended and unattended dimensions vary. Both same and different judgments should be facilitated when the irrelevant dimension is congruent and impeded when the irrelevant dimension is incongruent. Therefore, any interaction between ERP response to color congruency and shape congruency would occur at the time when the ERP component related to the response judgment was activated.

**Figure 2:**
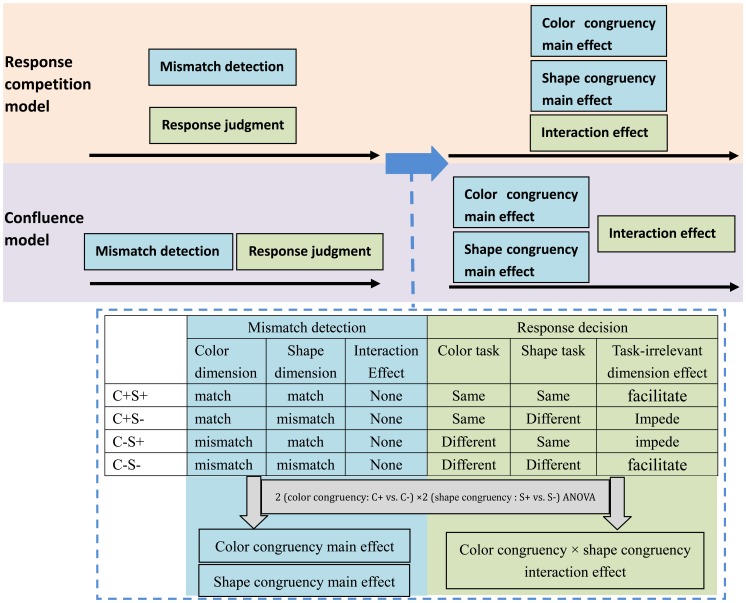
A graphic illustration of the predicted outcomes of the current study from a response competition model and a confluence model. In a response competition model, detection of the internal mismatch and the response judgment occur simultaneously, whereas in a confluence model mismatch detection occurs before response judgment. Both models predict that an internal mismatch is computed automatically regardless of whether it occurs in task-relevant or task-irrelevant dimensions. In the current study both models would predict color congruency and shape congruency main effects but no color congruency × shape congruency interaction at the mismatch detection stage. Both models would also predict facilitation (faster reaction times and corresponding ERP signal) of “same” responses when the information in the task irrelevant dimension was congruent and interference (slower reaction times and corresponding ERP signal) when it was incongruent. Additionally, both models also predict facilitation of “different” responses by a mismatch in the irrelevant dimension and interference by a match in irrelevant dimension. The models differ only in the time window in which the interaction should be observed. The Response Competition model predicts that the interaction effects would be observed in the same time window as the main effects. The confluence model predicts that the interaction effect should be observed after the main effects.

If the confluence model is correct, then the interaction between color congruency and shape congruency in the ERP data should appear after the main effects of congruency for either dimension, regardless of task. An alternative pattern of results in which the interaction effect occurs at the same time as the main effects of congruency in both dimensions would rather support a response competition model (see Figure 2).

## Materials and Methods

### 2.1 Participants

14 undergraduates (seven females) from the Liaoning Normal University of China were paid for their participation. All were right-handed with normal or corrected-to-normal vision and gave their informed written consent before participating in the study. This research was approved by the Research Ethics Committee of Liaoning Normal University of China and was conducted in accordance with the Declaration of Helsinki.

### 2.2 Stimuli

The stimuli consisted of images of two objects, presented simultaneously against a gray background on a 17- inch CRT monitor at a viewing distance of 70 cm. Each image consisted of one of five shapes (triangle, quadrangle, crisscross, round, pentagon) colored in one of five colors (red, yellow, blue, green, and pink. The two images were symmetrically positioned to the left and right of center screen. The display had a screen resolution of 800×600 and screen refresh rate of 85 Hz. The averaged visual angle of each figure was adjusted to 2.38°×2.38°. Based on whether or not two objects matched on color and shape dimensions, the stimulus pairs were divided into four types (Figure 1): C+S+, objects identical on color and shape; C+S−, objects matched on color, but not on shape; C−S+, objects matched on shape but not on color; C−S−, objects differed on both color and shape. The four types of stimulus pairs were randomly presented and had equal probability.

### 2.3 Procedure

Participants were seated in a dark, sound-attenuated room for the duration of the experiment. Each participant completed two sessions of comparison tasks under different instruction: (1) judge the similarity of the two objects on color (color task); (2) judge the similarity of the two objects on shape (shape task). In both conditions participants discriminated the two figures only on the basis of the instructed dimension and ignored the irrelevant dimension of the objects. The response keys were not counterbalanced because pilot testing found that the participants’ behavioral performance was worse when they were asked to respond ‘same’ with their right hand and ‘different’ with their left. This could reflect a similar response conflict to the observed in the Simon effect [29].

Each trial was performed in the following sequence. First, a fixation-cross appeared for a random duration ranging from 800 ms to 1,100 ms at the center of the screen. Then, the visual stimulus, consisting of two objects, was presented until a response was made. Participants were instructed to respond as quickly and as accurately as possible. During the experiment, participants were asked to keep head and eye movements to a minimum, while maintaining central fixation. A practice block was performed prior to each session. In the practice block, each participant completed 40 trials with10 trials for each condition. Order of trials within the two test sessions was randomized and each session consisted of 440 trials with110 trials for each condition and a break after every 88 trials. The session orders were counterbalanced among participants.

### 2.4 Electrophysiological (EEG) Recording and Analysis

A 129 lead geodesic sensor net measured brain and ocular scalp potential fields, with an evenly distributed sensor layout over the head surface and an inter-sensor distance of about 30 mm. Electrode impedance was kept below 50 kΩ. EEG data were recorded continuously with the vertex sensor as reference electrode. The EEG data were sampled at 500 Hz using an EGI amplifier (Electrical Geodesics, Inc., Eugene, Oregon). We re-referenced all signals off-line to an average of the mastoids and had them bandpass filtered (0.3–30 Hz). The EEG and electrooculogram (EOG) were epoched off-line into 800 ms periods including a 100 ms pre-stimulus baseline. We corrected *eye movement artifacts* with the Gratton–Coles- *Algorithm* using the EOG data [30]. In addition, we excluded the trials with artifacts (a voltage exceeding ±80 µV at any electrode location relative to baseline), and response errors from the analysis.

## Results

### 3.1 Behavioral Performance

**Figure 3:**
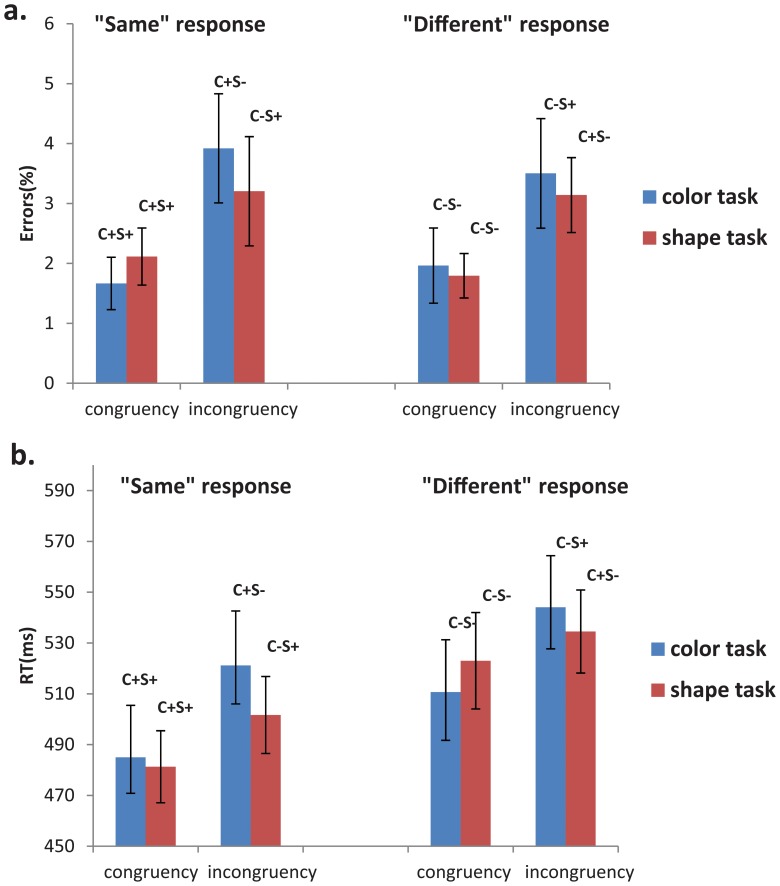
The error rates and mean response times in the shape task and the color task.

Trials with an RT of less than 200 ms or greater than two standard deviations from the participant’s mean were removed. For both tasks, mean error rates and RTs for the C+S+, C+S−, C−S+ and C−S−are shown in Figure 3. For errors, a 2 (task: color vs. shape) × 2 (judgment: same vs. different) × 2 (irrelevant dimension: congruent vs. incongruent) repeated measures ANOVAs demonstrated no task main effect [F(1,13) = .461, p>.05], no judgment main effect [F(1,13) = 0.2, p>.05], a significant irrelevant dimension main effect [F(1,13) = 21.54, p<.001], no interaction between task and irrelevant dimension [F(1,13) = 1.024, p>.05], no interaction between task and judgment [F(1, 13) = 0.002, p>.05], no interaction between judgment and irrelevant dimension [F(1, 13) = .016, p>.05], and no 3-way interaction [F(1, 13) = 0.933, p>.05].

An identical ANOVA on the reaction time data (RTs) showed no main effect of task [F(1,13) = .435, p>.05], but a significant judgment main effect [F(1,13) = 10.01, p<.01] (slower for ‘different’ than for ‘same’ judgments), a significant irrelevant dimension main effect [F(1,13) = 58.057, p<.001] and a significant interaction between task and irrelevant dimension [F(1,13) = 6.824, p<.05], but no interaction between task and judgment [F(1, 13) = 2.757, p>.05], or between judgment and irrelevant dimension [F(1, 13) = 1.272, p>.05] and no 3-way interaction [F(1, 13) = 0.519, p>.05]. Analysis of the simple main effects of the interaction between task and irrelevant dimension showed significantly slower RTs when the irrelevant dimension was incongruent in both the color task [F(1,13) = 43.6, p<.001] and the shape task[F(1,13) = 12.44, p<.01] with a significantly larger difference between the congruent condition and incongruent condition in the color task than in the shape task [F(1, 13) = −2.612, p<.05].

Overall response times were slower when the state (“same” or “different”) of the task-relevant dimension was incongruent with the state of task-irrelevant dimension, replicating previous behavioral studies on the SDJ task. Moreover, while the effect was significant in both tasks, it was bigger in the color task than in the shape task.

### 3.2. ERP Waveforms Analyses

**Figure 4:**
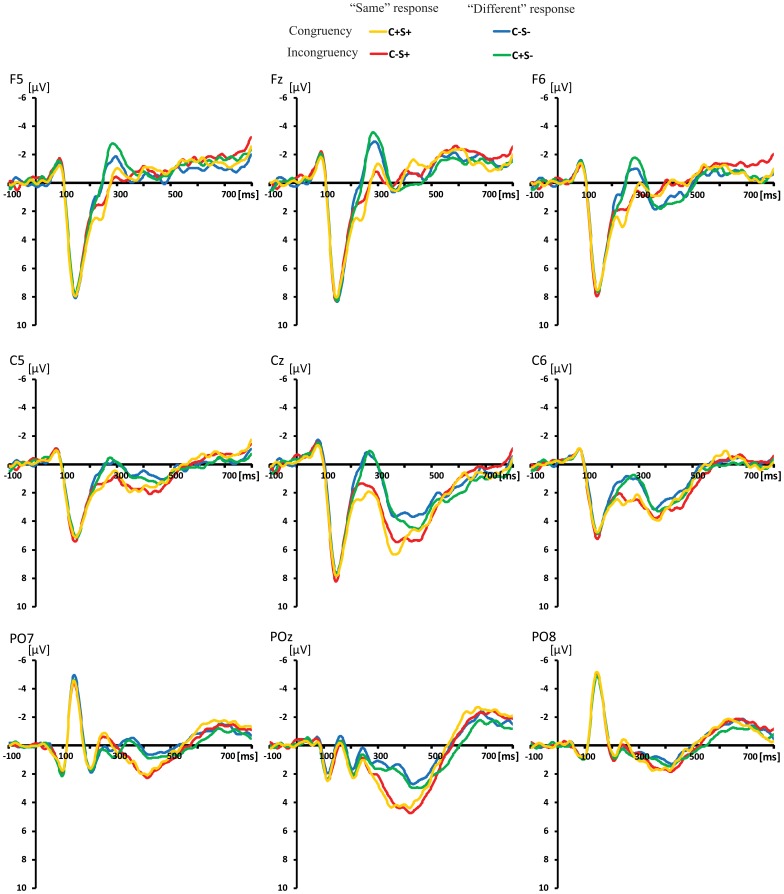
Grand average of the ERP data at the F5, Fz, F6, C5, Cz, C6, PO7, POz, PO8 sites for the C−S−, C−S+, C+S− and C+S+ conditions in the shape task.

**Figure 5:**
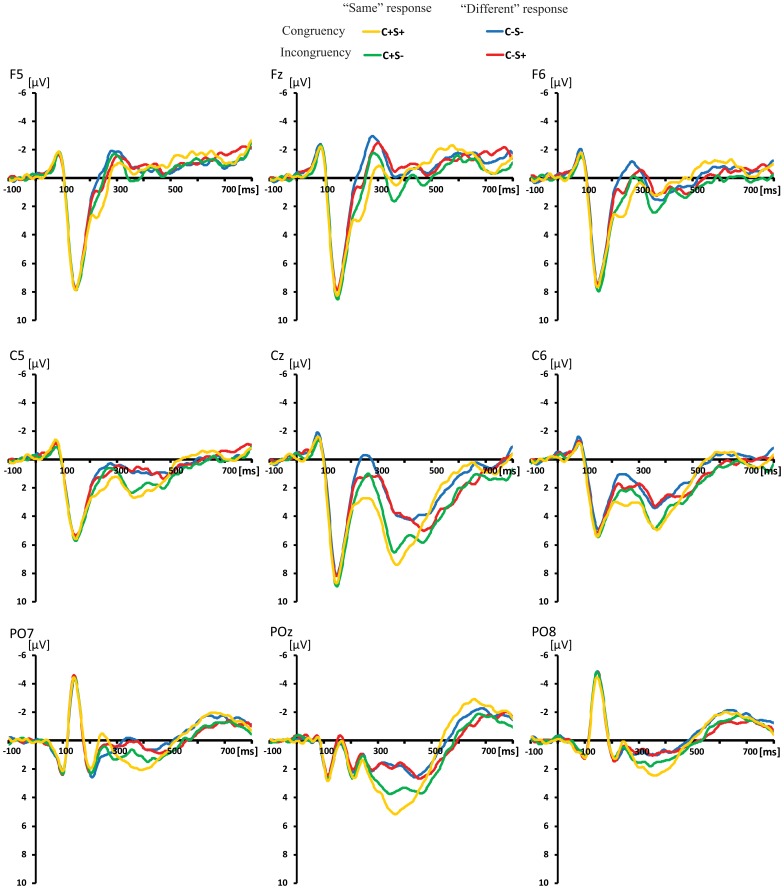
Grand average of the ERP data at the F5, Fz, F6, C5, Cz, C6, PO7, POz, PO8 sites for the C−S−, C−S+, C+S− and C+S+ conditions in the color task.

**Figure 6:**
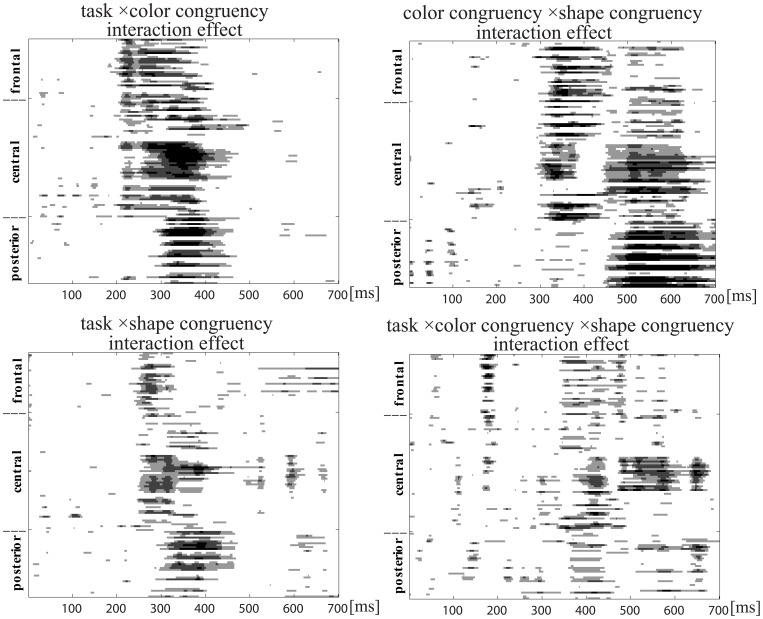
Statistical significance of 2(task: color and shape) × 2(color congruency: match and mismatch) × 2(shape congruency: match and mismatch) ANOVA task at each time point at each electrode.

**Figure 7:**
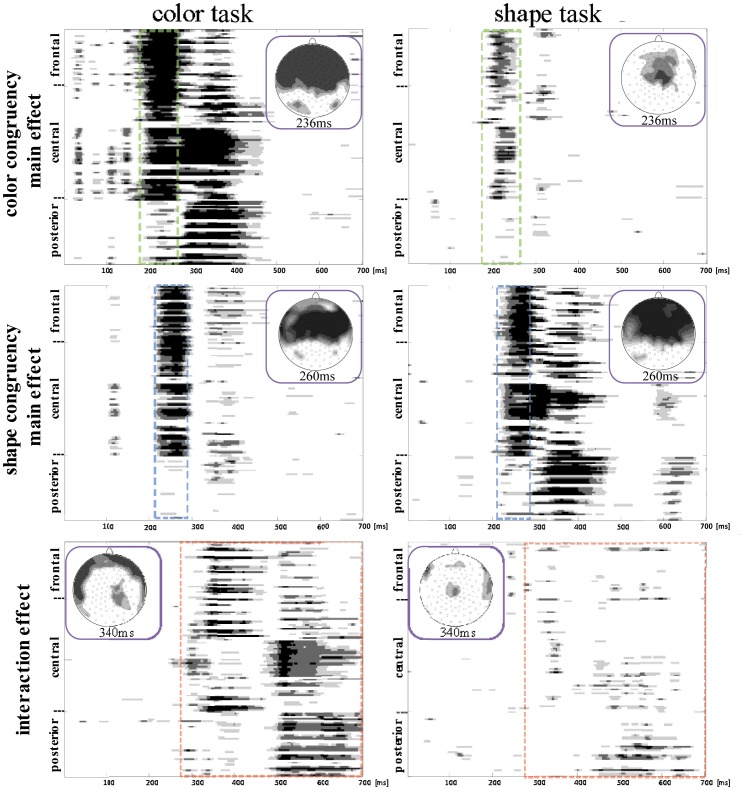
Statistical significance of 2(color congruency: match and mismatch) × 2(shape congruency: match and mismatch) ANOVA task at each time point at each electrode in the shape task and the color task.

The ERPs for the four stimuli types in each task were individually averaged. The ERP waveforms evoked by four conditions in the shape and color task are shown in Figure 4 and Figure 5. A 2 (task: color vs. shape) × 2 (color congruency: match vs. mismatch) × 2 (shape congruency: match vs. mismatch) repeated-measures ANOVA analysis at each time point at each electrode. there was an color congruency × shape congruency interaction effect, task ×color congruency interaction effect and task ×color congruency ×shape congruency interaction effect after approximately 180 ms post-stimulus (see Figure 6). Then, the effects of color congruency and shape congruency on the processing of stimuli were analyzed for each task using a 2 (color congruency: match vs. mismatch) × 2 (shape congruency: match vs. mismatch), repeated-measures analysis of variance (ANOVA), computed at each time point at each electrode. There was a significant main effect of color that firstly occurred at frontal and central sites at around 190–260 ms post-stimulus and was not modulated by shape congruency. The main effect of shape occurred at frontal and central sites at around 220–280 ms post-stimulus and was not modulated by color congruency. However, there was an interaction effect between color congruency and shape congruency occurring at the central sites after around 290 ms post-stimulus (see Figure 7).

Those spatio-temporal patterns that had a stable topography with a significant amplitude (p<0.01) at one electrode for at least 15 consecutive samples (30 ms) were considered as showing either significant main and/or interaction effects. On the basis of the ANOVA and the ERP topographical maps, those sites where the strongest activation was found were selected for further in-depth analysis. The Fz site was selected for further detailed analysis of ERP data during the 190–260 ms and 220–280 ms interval; the CPz site was selected for further detailed analysis of ERP data during the 290–430 ms interval.

#### 3.2.1 In-depth analysis of the color and shape main effects

The ERP waveforms showed that on both dimensions the mismatch information elicited more negative amplitude during the 190–280 ms interval. Furthermore, color and shape main effects were distributed over the frontal and central sites, resembling typical mismatch detection components (N270) in both scalp distribution and time phase. It appears that the more negative amplitudes for the C− and S− conditions during 190–280 ms relate to mismatch detection.

**Figure 8:**
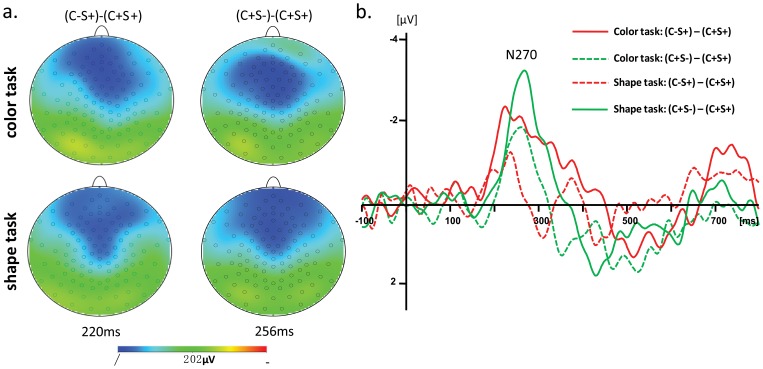
The scalp topography and difference waveforms for mismatch detection. a. The scalp topography for the difference waveforms between C−S+ and C+S+ at 236 ms and for the difference wave between C+S− and C+S+ at 260 ms. b. The difference waveforms between C−S+ and C+S+ and the difference waveforms between C+S− and C+S+ at the Fz site.

The difference waveforms between C−S+ and C+S+ conditions for the color and shape tasks were calculated to reflect the ERPs response to color mismatch, and the difference waveforms between C+S− and C+S+ conditions for the two tasks were used to reflect the ERPs response to shape mismatch (figure 8b). A N270 was observed in all difference waveforms during the 190–280 ms interval. The topographical map of N270 for color mismatch at 236 ms post-stimulus and for color mismatch at 260 ms post-stimulus showed a distribution in the frontal-central scalp areas (Figure 8a), which was similar with the topographical map of color and shape main effect (Figure 7). Thus, the Fz site was selected for statistical analysis of N270. The peak latencies and amplitude of N270 were analyzed through a 2 (attention: attended vs. unattended) × 2 (target dimension: color vs. shape) repeated measures ANOVA. For color judgments, the data on the color task was attended, and that from shape task was unattended. For the shape dimension, the data from the shape task was attended, and that from the color task was unattended. For the N270 amplitude, there was a significant attention main effect [*F*(1, 13) = 20.83, *p*<.001] and a target dimension main effect [*F*(1, 13) = 5.97, *p*<.05]. A post-hoc analysis showed that the N270 amplitude for the mismatch detection was larger in the attended than in the iunattended condition, and was larger for the shape target dimension than the color target dimension. For the N270 latency, there was a significant target dimension main effect [*F*(1, 13) = 16.52, *p*<.01]. A post-hoc analysis showed that the N270 latency for the mismatch detection in the shape dimension occurred later than in the color dimension.

#### 3.2.2 In-depth analysis of the interaction between color congruency and shape congruency

**Figure 9:**
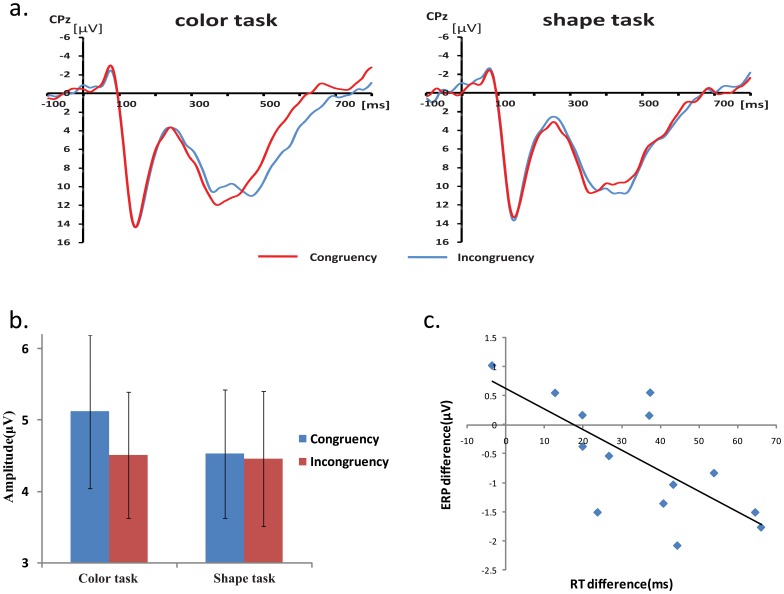
ERP waveform analyses result for state congruency. a. The ERP waveforms for the stimuli with congruent and incongruent response states (“same” or “different”) of the task-relevant and -irrelevant dimensions at CPz. b. The mean amplitude of the ERP responses to the stimuli with congruent and incongruent response states (“same” or “different”) of the task-relevant and -irrelevant dimensions during the 290–430 ms interval at CPz. c. The correlation between the ERP amplitude difference and RT difference revealed between the incongruent states and congruent states in the color task.

In both tasks, there was an interaction between color congruency and shape congruency at central sites after approximately 290 ms post-stimulus. This interaction lasted for two time windows: 290–430 ms, 500–700 ms. Since mean RTs for all conditions were less than 540 ms, the interaction effect in the 500–700 ms window occurred after the keypress response, so is unrelated to task processing and is not considered further. The topographical map of the interaction during the 290–430 ms interval showed a distribution in the parietal scalp areas (Figure 7). Moreover, the CPz site was selected for post-hoc analysis of ERP data during the 290–430 ms interval. The ERP waveform (S−C−+S+C+)/2 was used to reflect the ERPs response to the stimuli with the congruent response state (same different) between the task-irrelevant dimension and task-relevant dimension, and the ERP waveform (S+C−+S−C+)/2 was used to reflect the ERPs response to the stimuli with the incongruent response state (Figure 9a). The mean amplitude for each condition (Figure 9b) over the time interval of 290–430 ms were analyzed in a 2 (task type: color vs. shape) × 2 (state congruency: congruent vs. incongruent) ANOVA. There was a significant interaction between task type and state congruency [*F*(1, 13) = 9.14, *p*<.01]. A post-hoc analysis showed a significantly larger mean amplitude for congruent states [*t*(1,13) = 2.833 *p*<.05] in the color task, but not in the shape task [*t*(1,13) = .681, *p*>.05].

In addition, both the ERP amplitude difference and RT difference between the incongruent states and congruent states in the color task were entered into a correlation analysis. There was a significant negative correlation between ERP and RT differences [*R* = −0.711, *p*<0.01] (Figure 9c), indicating that more negative ERP amplitudes are related to response delays caused by the conflict between congruency/incongruency in the color and shape dimensions.

## Discussion

This study adds to our understanding of the neural processes that underlie making “same”-“different” judgments on a task-relevant dimension about two simultaneously presented images that are also either the same or different on a task-irrelevant dimension. To investigate the congruency effects on those judgments, we assessed both behavioral and ERP measures when the congruency of state (“same” or “different”) between two images in the task-relevant and task-irrelevant dimensions were manipulated. The findings of this study replicate previous behavioral studies, and extend previous EEG studies, as well as providing support for the hypothesis that this type of information is processed in parallel.

RTs for “same” or “different” judgments on the task-relevant dimension were slower when the match/mismatch of objects in the task-irrelevant dimension was incongruent with the task-relevant dimension compared to when it was congruent. These results are consistent with previous behavioral findings of a significant effect of the task-irrelevant dimension on the task-relevant dimension [8], [9]. In addition, we found a greater delay when the task-relevant dimension was color than when it was shape. This is discussed together with the ERP results below.

In the ERP analysis, beyond 190 ms post-stimulus, a more negative ERP waveform was elicited over the fronto-central scalp region by the S−C−, S−C+ and S+C− conditions compared to the baseline S+C+ condition in both color and shape tasks. This negative component was similar to the N270 component previously observed in sequential matching task [19], [22]. Converging evidence suggests that the N270 is related to the detection of mismatch between two objects [14]–[16], [22]. Thus, more negative ERP waveforms for S−C−, S−C+ and S+C− conditions can be attributed to mismatch detection. In addition, the color congruency main effect in the time windows 190–260 ms occurred before the shape congruency main effect in the time windows 220–280 ms for both color and shape tasks, without a significant interaction between color congruency and shape congruency. This indicates that the negative component at 190–260 ms related to color mismatch detection, and the negative component in the time windows 220–280 ms was related to shape mismatch detection. Since the main effects of color and shape congruency were still observed when they were task-irrelevant, these results suggest that the detection of mismatch in the task-irrelevant dimension is automatically processed implicitly. Nevertheless, the match/mismatch on the relevant stimulus dimensions lead to bigger ERP effects than that on irrelevant ones, so attention to the task-relevant dimension may attenuate the automatic processing of task-irrelevant dimensions.

The lag in the timing of the shape congruency effect compared to the color congruency effect implies sequential detection of a mismatch in color and shape dimensions, supporting a serial processing model. However, in a serial process model, paying attention to different dimensions should prompt participants to prioritize the detection of mismatch on the attended dimension. In the present experiment, the order of appearance of the main effect of shape and color congruency on ERPs did not vary with task, indicating that attention to either dimension did not change the order of mismatch detection on the two dimensions. So the mismatch between color and shape dimensions appears to be detected automatically and independent of which dimension is attended.

The difference between the timing of detection of mismatch in the color and shape dimensions might result from differential speed of information processing on the two dimensions. This in turn implies either that processing shape information requires more time, compared to processing color.

Furthermore, the interaction between color congruency and shape congruency occurred after about 290–430 ms post-stimulus in both tasks. Given the reciprocal facilitation of responses on both tasks when the irrelevant dimension was congruent and the interference on both tasks when the irrelevant dimension was incongruent, the interaction between color congruency and shape congruency suggests that there is an ERP component associated with the response. This hypothesis was further supported by the significant correlation between the ERP amplitude difference and the RT difference between the incongruent and congruent states in the task-relevant and –irrelevant dimensions. The late occurrence of the interaction, about 290 ms post-stimulus (later than the ERP for shape and color congruency), suggests that the response selection occurred only after the detection of mismatch had been completed, which provides support for the confluence model [12].

A previous study in which participants were asked to judge whether the combined attributes of color and shape were the same or different for sequential presented pairs reported that two negative peaks (N270 and N400) were recorded in the ERP in the conjunction-mismatch condition (with a mismatch in both color and shape dimensions) in a dual feature matching task [22]. Furthermore, the amplitude of the N270 for the conjunction-mismatch was smaller in the dual feature-matching task than in the color or shape-matching task. Those authors proposed that the N270 was related to mismatch detection for one feature and the N400 was related to mismatch detection for the other feature, suggesting that detection of color and shape mismatch occurred sequentially.

In contrast, our findings suggest that the detection of mismatch in color and shape dimensions occurs automatically and in parallel. These conflicting findings may reflect the fact that dual feature matching requires participants to decide whether or not the mismatch is presented in both color and shape dimensions. That task, compared to the SDJ task used here, involves an additional logical step based on comparison of the outputs of detection of mismatch of color and shape dimensions. Therefore the N400 may relate to processing the latter logical judgment instead of processing mismatch detection. The reduction of N270 amplitude in dual feature matching may result from reduced attention for a given dimension caused by increased attentional load.

Both RT and ERP results revealed similar asymmetries in that the interaction between congruence and the nature of the relevant task (color or shape) was greater for the color task than for the shape task. It has been also found that a shape mismatch elicited a much more negative N270 than a color mismatch in the task-irrelevant condition [22]. The authors speculated that shape mismatch processing occurred automatically regardless of task-relevance. Our findings lend support to this interpretation, with larger ERP amplitude differences for the detection of shape mismatches than for color mismatches, at the same time as a larger facilitation effect for congruent dimensions when color was the attended dimension.

An alternative explanation of these results might be provided by a load theory of attention. The load theory of attention suggests that both early perceptual selection mechanisms and late selection mechanisms of cognitive control could affect the processing of distractors [31]. A perceptual selection mechanism reduces the perceptual processing of distractors when perceptual processing capacity is fully taken up by relevant stimuli and a cognitive control mechanism reduces interference from perceived distractors as long as cognitive control functions are available to maintain processing priorities. The mismatch detection is a late cognitive processing. Thus, according to the load theory of attention, the color task could involve higher load on cognitive control functions than shape task. These could result from that making “same-different” judgments for color stimuli need more working memory than for shape stimuli, or the color mismatch processing is more difficult than shape mismatch processing.

It might also have been the case that the set of colors used in the present experiment may have been selectively more different from each other than the set of shapes, although both are considered to be perceived categorically by adult [32], [33].

## Conclusion

The present study used ERP to investigate how “same-different” judgments on the task-relevant dimension would be affected by the state (“same” or “different”) of the objects in the task-irrelevant dimension. Findings from this study indicated that mismatch detection in task-relevant and task-irrelevant dimensions could be processed automatically and independently. The effect of task-irrelevant dimension on the response to task-relevant dimension occurred after mismatch detection had occurred, providing a strong evidence for the confluence model.
