# Correction: Neural Processes Underlying the“Same”-“Different” Judgment of Two Simultaneously Presented Objects- An EEG Study

**DOI:** 10.1371/annotation/707d3fe3-ac97-434b-a7c7-803895d4cab0

**Published:** 2014-01-16

**Authors:** Ruiling Zhang, Zhonghua Hu, Debi Roberson, Lingcong Zhang, Hong Li, Qiang Liu

The name of the third author is incorrect. The correct name is: Debi Roberson. 

The correct Citation is: Zhang R, Hu Z, Roberson D, Zhang L, Li H, et al. (2013) Neural Processes Underlying the“Same”-“Different” Judgment of Two Simultaneously Presented Objects- An EEG Study. PLoS ONE 8(12): e81737. doi:10.1371/journal.pone.0081737. 

The correct abbreviation of the third author's name in the Author Contributions statement is: DR.

